# Preventing, identifying, and managing medication-related osteonecrosis of the jaw: a practical guide for nurses and other allied healthcare professionals

**DOI:** 10.1007/s00520-020-05440-x

**Published:** 2020-04-19

**Authors:** Lawrence Drudge-Coates, Tim Van den Wyngaert, Morten Schiødt, H. A. M. van Muilekom, Gaston Demonty, Sven Otto

**Affiliations:** 1 Department of Urology, King’s College Hospital NHS Foundation Trust, Denmark Hill, London, SE5 9RS UK; 2 Department of Nuclear Medicine, Antwerp University Hospital, Edegem, Belgium; 3 Faculty of Medicine and Health Sciences, University of Antwerp, Antwerp, Belgium; 4 Department of Oral and Maxillofacial Surgery, Rigshospitalet, Copenhagen, Denmark; 5 Department of Clinical Epidemiology, Aarhus University Hospital, Aarhus, Denmark; 6 Department of Urology, Antoni van Leeuwenhoek Hospital–Netherlands Cancer Institute, Amsterdam, Netherlands; 7 Medical Development, Amgen (Europe) GmbH, Rotkreuz, Switzerland; 8 Department of Oral and Maxillofacial Surgery, Ludwig-Maximilians-University of Munich, Munich, Germany

**Keywords:** Awareness, Clinical management, Counseling, Jaw necrosis, MRONJ

## Abstract

**Background:**

Medication-related osteonecrosis of the jaw (MRONJ) is an infrequent, but potentially serious, adverse event that can occur after exposure to bone-modifying agents (BMAs; e.g., bisphosphonates, denosumab, and antiangiogenic therapies). BMAs are typically used at higher doses to prevent skeletal-related events in cancer patients and at lower doses for osteoporosis/bone loss. MRONJ can cause significant pain, reduce quality of life, and can be difficult to treat, requiring a multiprofessional approach to care.

**Methods:**

We reviewed the literature and guidelines to summarize a practical guide on MRONJ for nurses and other allied healthcare professionals.

**Results:**

While there is a risk of MRONJ with BMAs, this should be considered in relation to the benefits of treatment. Nurses and other allied healthcare professionals can play a key role alongside physicians and dentists in assessing MRONJ risk, identifying MRONJ, counseling the patient on the benefit–risk of BMA treatment, preventing MRONJ, and managing the care pathway of these patients. Assessing patients for MRONJ risk factors before starting BMA treatment can guide preventative measures to reduce the risk of MRONJ. Nurses can play a pivotal role in facilitating multiprofessional management of MRONJ by communicating with patients to ensure compliance with preventative measures, and with patients’ physicians and dentists to ensure early detection and referral for prompt treatment of MRONJ.

**Conclusions:**

This review summarizes current evidence on MRONJ and provides practical guidance for nurses, from before BMA treatment is started through to approaches that can be taken to prevent and manage MRONJ in patients receiving BMAs.

**Electronic supplementary material:**

The online version of this article (10.1007/s00520-020-05440-x) contains supplementary material, which is available to authorized users.

## Introduction

Medication-related osteonecrosis of the jaw (MRONJ) is an infrequent, but potentially serious, adverse event associated with bone-modifying agents (BMAs; principally bisphosphonates and denosumab) and antiangiogenic therapies [1–3]. MRONJ has also been reported with tyrosine-kinase inhibitors, mechanistic target of rapamycin inhibitors, *BRAF* inhibitors, immune checkpoint inhibitors, and cytotoxic chemotherapy [3]. BMAs are typically used at higher doses in patients with cancer to prevent skeletal-related events (SREs) [4], a term that includes the following major complications of bone disease related to tumors: fractures, orthopedic intervention, radiotherapy, and spinal cord compression [5, 6]. At lower doses, BMAs are used to treat osteoporosis and treatment-induced bone loss [4, 7].

The risk of MRONJ is greater in patients receiving high-dose BMAs (usually for metastatic bone disease) than in those receiving low-dose BMAs (usually for osteoporosis), with a reported incidence of 1–9% [2] and 0.10% [8], respectively. Indeed, 90% of MRONJ cases occur in patients with cancer receiving high-dose BMAs [9].

The pathogenesis of MRONJ has not yet been fully elucidated, but it likely involves several factors, most importantly infection/inflammation, but also impaired bone repair, suppression of osteoclast activity, altered immunity, soft tissue toxicity, and angiogenesis inhibition after exposure to BMAs [10]. The oral microbiome and dental infections are thought to be central to MRONJ development [11, 12].

MRONJ can cause significant pain, reduced quality of life, and can be difficult to treat [1–3], requiring a multiprofessional approach to care. Comprehensive reviews aimed at physicians [13] and dentists [4] have been published. This review is aimed at nurses and other allied healthcare professionals who may encounter patients with or at risk of MRONJ. Early detection of MRONJ can be challenging but is important. Nurses should be vigilant about identifying risks and symptoms, help patients consider the benefit–risk of BMAs, take steps to prevent MRONJ, and facilitate multiprofessional treatment of MRONJ when needed.

## Diagnosis of MRONJ and patient identification

**Fig 1:**
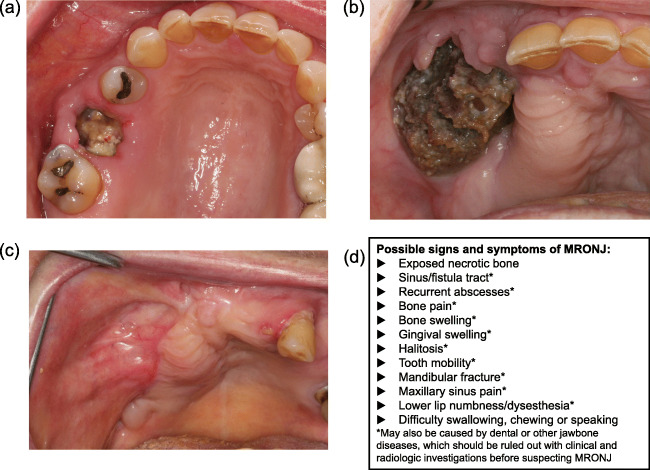
Images of MRONJ (**a**) after tooth extraction of upper first molar in a 55-year-old woman with lung cancer treated with bevacizumab (VEGF inhibitor), (**b**) another patient with MRONJ before and (**c**) after treatment (i.e., healthy tissue), and (**d**) the possible signs and symptoms of MRONJ (modified from [17]) *MRONJ*, medication-related osteonecrosis of the jaw; *VEGF*, vascular endothelial growth factor. Images courtesy of Morten Schiødt

According to the American Association of Oral and Maxillofacial Surgeons (AAOMS), a patient is considered to have MRONJ if they meet all the criteria in Table 1 [14]. An example of exposed MRONJ is shown (Fig. 1a). However, up to one-quarter of patients experiencing MRONJ present with a non-exposed form [15], which should be recognized and treated similarly to exposed MRONJ [16]. Patients presenting with MRONJ with non-exposed bone were not historically formally diagnosed with the condition [15, 17, 18]. This prompted the AAOMS to include “bone that can be probed through a fistula” as a criterion for MRONJ in 2014 [14]. The AAOMS requirement of at least 8 weeks’ observation for MRONJ manifestation may not be necessary [17]. These points are important to keep in mind when assessing for MRONJ.

**Table 1:** AAOMS 2014 criteria for diagnosing MRONJ and MRONJ stages [14]

Criteria for diagnosis of MRONJ	
1. Current or previous treatment with BMAs or antiangiogenic agents 2. Exposed bone or bone that can be probed through a fistula, situated within or outside the mouth, in the maxillofacial region that has persisted for longer than 8 weeks 3. No history of radiation therapy to the jaws or obvious metastatic disease to the jaws	
MRONJ stages	
• “At risk” includes patients who have been treated with BMAs but who have no apparent necrotic bone	
• Stage 0 includes patients with no clinical evidence of necrotic bone but who have non-specific symptoms or clinical/radiographic findings	
• Stage 1 includes patients with exposed and necrotic bone, or fistulae that probe to bone, who are asymptomatic with no evidence of significant adjacent or regional soft tissue inflammation or infection	
• Stage 2 includes patients with exposed and necrotic bone, or fistulae that probe to bone, associated with infection, as shown by pain and adjacent or regional soft tissue inflammatory swelling, with or without purulent drainage	
• Stage 3 includes patients with exposed and necrotic bone, or fistulae that probe to bone, associated with pain and infection, and at least one of the following: (1) pathologic fracture, (2) an extra-oral fistula, (3) an oral-antral fistula, or (4) radiographic evidence of osteolysis extending to the inferior border of the mandible or the floor of the maxillary sinus	

*AAOMS*, American Association of Oral and Maxillofacial Surgeons; *BMA*, bone-modifying agent; *MRONJ*, medication-related osteonecrosis of the jaw

An example of MRONJ before and after treatment (i.e., healthy tissue) is shown (Fig. 1b, c). MRONJ is staged from “at risk” to stages 0–3 (Table 1) [14]. The signs and symptoms of MRONJ, which can result in difficulties with chewing, eating, and speaking, may include the following: exposed necrotic bone; sinus fistula; signs of infection (e.g., recurrent abscesses, localized bone pain, and bone/gum swelling); bad breath; loose teeth; fracture of the jaw; maxillary sinus pain; and lower lip/chin numbness/heaviness (Fig. 1d) [19]. With the exclusion of exposed necrotic bone, these signs/symptoms may also be caused by dental or other jawbone diseases, which should be excluded before suspecting MRONJ [17]. Jaw pain was reported in over three-quarters of patients who developed MRONJ in an integrated analysis of data from 5723 patients with bone metastases associated with solid tumors/multiple myeloma (MM) in three phase 3 trials of high-dose denosumab or zoledronic acid [20]. Oral infections associated with tooth extractions were reported for nearly one-half of patients with MRONJ. The most common site of MRONJ was the mandible (less frequently in the maxilla). In a small number of patients (< 5%), MRONJ was observed in both locations [20].

To enable early detection of MRONJ, nurses can proactively ask “Are there any areas of soreness, numbness or signs of damage in your mouth?” Patients should be prompted to report symptoms such as difficulty with eating and/or speaking [19]. If in doubt, nurses should discuss possible signs and symptoms of MRONJ with the treating physician/dentist, and the patient can be referred to an oral and maxillofacial surgeon or an experienced oral oncology center [21].

## Risk factors for MRONJ

The Multinational Association of Supportive Care in Cancer/International Society of Oral Oncology/American Society of Clinical Oncology (MASCC/ISOO/ASCO) Clinical Practice Guideline (2019) advises on best practice in the prevention and management of MRONJ in patients with cancer, based on expert opinion and evidence from a comprehensive literature review [2]. This guidance lists risk factors for the MRONJ development; these are summarized in Table 2. Key studies, which elucidated MRONJ risk factors, are discussed in the following sections.

**Table 2:** Risk factors for MRONJ [2]

MASCC/ISOO/ASCO Clinical Practice Guideline significant risk factors for MRONJ	
• BMA treatment	
• High-dose BMA	
• Longer duration of BMA therapy	
• Dental extraction and other oral surgical procedures	
• Periodontal disease/infection (treatment of infection reduces risk)	
• Denture use	
• Tobacco use	
• Angiogenesis inhibitors	
• Diabetes	
MASCC/ISOO/ASCO Clinical Practice Guideline factors possibly affecting risk of MRONJ	
• Chemotherapy	
• Corticosteroids	
• Cancer site (for example, breast cancer or multiple myeloma)	
• Renal disease	
• Erythropoietin therapy	
• Hypothyroidism	
• Being female	
• Being Caucasian	
• Older age	

*BMA*, bone-modifying agent; *MASCC/ISOO/ASCO*, Multinational Association of Supportive Care in Cancer/International Society of Oral Oncology/American Society of Clinical Oncology; *MRONJ*, medication-related osteonecrosis of the jaw

## MRONJ risk factors with high-dose BMAs

Two robust studies evaluated MRONJ risk factors with high-dose BMAs: the previously mentioned integrated analysis of data from 5723 patients treated with high-dose denosumab or zoledronic acid in three phase 3 trials [20] and the prospective SWOG0702 trial of approximately 3500 patients with metastatic bone disease receiving zoledronic acid [22].

### Integrated analysis

MRONJ incidence was 1.8% with denosumab and 1.3% with zoledronic acid (median time on study ~ 1 year for both arms) [20]. Tooth extractions and coinciding oral infections occurred in 61.8% and 48.3% of patients with MRONJ, respectively. Compared with patients without MRONJ, a greater proportion of those with MRONJ received corticosteroids or antiangiogenic agents (however, only a small number of patients with MRONJ were exposed to antiangiogenic agents). Similar percentages of patients with and without MRONJ had anemia, diabetes, or received chemotherapy. MRONJ incidence increased with time on therapy and thus cumulative BMA exposure.

### SWOG0702 trial

In the SWOG0702 trial, the cumulative incidence of MRONJ at 3 years was 2.8% overall [22]. Analysis by cancer type demonstrated a higher 3-year risk in MM (4.3 versus 2.9% for prostate cancer, 2.7% for lung cancer, and 2.4% for breast cancer). A zoledronic acid dosing interval of every 3–4 weeks increased the MRONJ risk nearly five times compared with a less frequent dosing regimen (e.g., every 12 weeks). Prior oral surgery nearly doubled the risk, and being a current smoker more than doubled the risk. However, two smaller trials did not show a difference in MRONJ risk between 4 weekly and 12 weekly dosing, illustrating the difficulty in studying the epidemiology of infrequent adverse events in clinical trials that are powered only for efficacy outcomes [23, 24]. The MRONJ risk in SWOG0702 was reduced by approximately one-third in patients with more than the median number of mandibular teeth, and by approximately half in those with more than the median total number of teeth [22]. Tooth loss is often caused by infection (e.g., marginal/apical periodontitis); we hypothesize that the higher incidence of MRONJ in patients with fewer teeth is likely due to infection as an underlying factor. Tooth loss may also be linked to denture use, which approximately doubled the risk of MRONJ [22], likely due to denture-related traumatic ulcers and subsequent exposed bone. Further data from this trial are eagerly awaited.

### Other trials examining risk factors associated with high-dose BMAs

Cumulative BMA dose may be a MRONJ risk factor, as may the type of cancer. Data from two phase 3 studies in breast cancer and prostate cancer showed that the incidence of MRONJ was 1.1 per 100 patient-years during the first year of denosumab treatment, increasing to 4.1 per 100 patient-years in subsequent years [25]. Another phase 3 study in patients with newly diagnosed MM reported a patient-year adjusted incidence of MRONJ of 2.0 per 100 patient-years during the first year of treatment, 5.0 in the second year, and 4.5 per year thereafter [26]. A retrospective study assessed the incidence of, and risk factors associated with, MRONJ in 120 patients with MM treated with bisphosphonates following high-dose chemotherapy and autologous stem cell transplantation; 23 (19%) patients developed MRONJ [27]. Patients with MM therefore deserve particular attention for MRONJ prevention and early detection. The incidence of MRONJ in the retrospective study was associated with rheumatic disease, recent dental manipulations, and elevated C-reactive protein levels (suggesting infection). Previous bisphosphonate exposure, duration of bisphosphonate therapy, cumulative dose of bisphosphonate, and the type of bisphosphonate administered were identified as MRONJ risk factors [27].

## MRONJ risk factors with low-dose BMAs

Identifying risk factors for MRONJ in patients with osteoporosis receiving low-dose BMAs is challenging, as evidence is limited due to the very low frequency of MRONJ. In the FREEDOM extension study, which involved 3591 post-menopausal women with osteoporosis, there were only 12 cases of MRONJ after up to 7 years of low-dose denosumab (plus 3 years of denosumab in the main FREEDOM study) [28]. Nearly half the women had undergone at least one invasive oral procedure, and the incidence of MRONJ was higher in these women compared with those that did not (0.68 versus 0.05%). A systematic review identified 680 cases of MRONJ in patients with osteoporosis from 44 studies [29]. Duration of anti-absorptive drug treatment was found to be a MRONJ risk factor, as was concomitant corticosteroid/immunosuppressive treatment. Dental extractions followed by dentoalveolar surgery were the most frequent events prior to MRONJ, occurring in 48.5% and 21.1% of patients, respectively. In these cases, MRONJ was likely triggered by an underlying chronic local infection, which resulted in dental extraction, rather than the procedure [29]. Infection may develop as a consequence of the dental procedure, when bacteria gain access to bone because extraction sockets are left open to heal spontaneously or when wound healing is incomplete [30]. Alternatively, infection may be the reason for the tooth extraction. Indeed, a retrospective study of patients with osteoporosis and/or malignant tumors treated with bisphosphonates undergoing tooth extraction or surgical tooth removal revealed that in 69% of cases, osteomyelitic or osteonecrotic changes were already present at the time of the extraction [30].

## Patient risk assessment

To assess MRONJ risk in patients before starting a BMA, please see Online resource 1; Supplementary data 1 for questions to ask your patients. If the patient answers “Yes” to any of these questions, they have an increased risk of developing MRONJ. Cumulative exposure to BMA is a key risk factor; this includes the dose per treatment, frequency of treatments, and duration of treatment, as well as the potency, but not the route of administration. Studies analyzing administration route as a risk factor tend to be confounded by the fact that denosumab is administrated subcutaneously, regardless of dose, whereas low-dose bisphosphonates tend to be oral and high-dose bisphosphonates tend to be intravenous [4, 31]. Generally, high-dose BMA prescribed for patients with metastatic cancer is associated with a higher risk of MRONJ compared with low-dose therapy for osteoporosis [17]. In addition to the dose per administration, the intensity of dosing may be up to 12 times more frequent in the setting of bone metastases compared with osteoporosis. Therefore, the dose, frequency, and duration of BMA treatment are included as additional considerations, as is age. Online resource 1; Supplementary data 2 provides a flow chart to assess MRONJ risk. Using this chart, patients receiving high-dose BMAs would automatically be considered to be at elevated risk.

Nurses can play a unique role in translating the benefit–risk ratio of BMAs to patients who may be concerned about the risk of MRONJ. Nurses can do this by educating the patient on the potential advantages of treatment. While this may appear trivial, it is well documented that the patient’s perception of risk carries more weight in decision-making than the probability of harm, even if the benefits are clear for oncologic and osteologic indications [32]. This benefit–risk will vary from patient to patient, depending on the individual’s risk to develop an SRE (e.g., disease extent, location, and activity), the presence of risk factors for MRONJ, and how a patient perceives having an SRE or developing MRONJ.

In the osteoporosis setting, the FREEDOM study of low-dose denosumab in 4074 post-menopausal women showed an MRONJ incidence rate of 0.05 per 100 patient-years (i.e., 5 cases of MRONJ for every 10,000 years of treatment) [33]. In comparison, the rate of non-vertebral fractures with 3 years of placebo was 2.65 per 100 patient-years (i.e., 265 fractures per 10,000 years) whereas patients treated with 3 years of denosumab had 2.15 fractures per 100 patient-years, and this was significantly reduced to 1.53 per 100 patient-years with a further 4 years of denosumab treatment.

In terms of explaining benefit–risk to the patient, it could be said that “The chance of preventing a SRE using BMA treatment is greater than the risk of developing MRONJ as a result of treatment, and there are steps that can be taken to reduce the risk of MRONJ even further.” Statements based on average treatment effects and risks can be derived from clinical trial data and may further assist nurses in communicating the magnitude of benefit–risk to patients, ultimately leading to more informed decisions. For example, a woman with breast cancer that has spread to the bones, who considers the harm that comes from a bone complication to be equal to the harm from MRONJ, could be told that “On average, you are 17 times more likely to derive benefit than harm during the first two years of denosumab treatment, compared with choosing no bone-directed treatment” (Online resource 1; Supplementary data 3).

As patients often give risk more weight than benefit, patient decision aids, such as handouts, may help inform patients on the benefits and harms associated with different therapy options, aiding in shared decision-making [32, 34]. For example, the Scottish Dental Clinical Effectiveness Programme has published dental clinical guidance [35], which includes a section on “Points to Cover During MRONJ Risk Discussion,” and provides an illustrated figure for explaining risk to patients (Online resource 1; Supplementary data 4). Tools are available from other therapy areas, for example, The Royal College of Obstetricians and Gynaecologists produced a patient leaflet called “Understanding how risk is discussed in healthcare,” which can be found online [36]. Similar aids could be created as valuable tools for oncology nurses when discussing treatment options with patients at risk of MRONJ.

## Prevention of MRONJ and implications for nurses and allied healthcare professionals

**Fig. 2:**
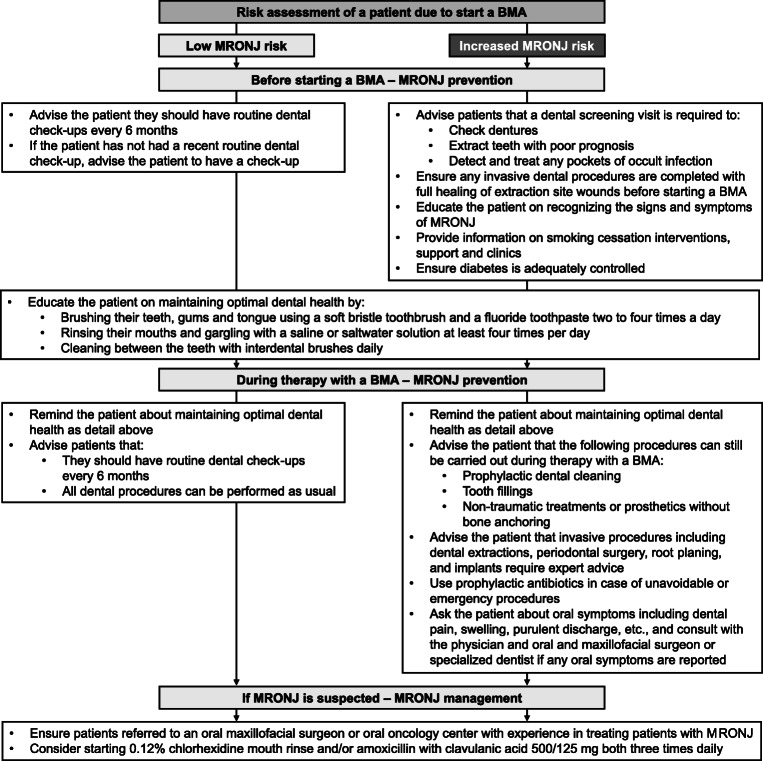
Approach for preventing and managing MRONJ. *BMA*, bone-modifying agent; *MRONJ*, medication-related osteonecrosis of the jaw

In addition to counseling the patient who may have concerns about MRONJ, working with the patient to prevent the condition is a key. Nurses can provide clear explanations, information, and education on MRONJ risks and the preventative steps to reduce this risk. Figure 2 details approaches for preventing and managing MRONJ based on the assessment of MRONJ risk determined by the answers to the questions/flowchart in Online resource 1; Supplementary data 1/2. Nurses can empower patients to be part of the process of preventing MRONJ through following best practice. Ensuring patient compliance with MRONJ preventative measures and any subsequent treatment is vital and requires a level of understanding from the patient.

MRONJ prevention should be started prior to BMA treatment. For low-risk patients, the same recommendations on preventative dental visits and maintaining optimal dental health for the general population apply [1, 2, 37]. For patients at increased risk of MRONJ, the MASCC/ISOO/ASCO Clinical Practice Guideline considers modifiable risk factors to be poor dental care, invasive dental procedures, poorly fitting dentures, high blood sugar, and smoking [2]. Risk reduction strategies to prevent MRONJ before and during denosumab/bisphosphonate treatment include premedication dental evaluation, maintaining optimal oral hygiene, and having regular dental check-ups [1, 2, 14, 38]. An example letter for the patient’s dentist, explaining that the patient will be starting a BMA and a dental evaluation is required, as well as information sheets for the dentist, is included in Online resource 1; Supplementary data 5. Dental evaluation before starting BMAs has been shown to significantly reduce the MRONJ incidence [39]. A systematic review of risk-reductive dental strategies for MRONJ in six studies of 2332 cancer patients found that preventative dental measures significantly decreased MRONJ incidence by approximately three-quarters versus control groups [40]. Ensuring adequate control of diabetes and providing information on smoking cessation are also important when addressing modifiable risk factors prior to starting a BMA [2].

MRONJ prevention should continue during BMA treatment. It is important to continue to remind patients about preventative dental visits and maintaining optimal dental health. Nurses should stress the importance of monitoring for and reporting symptoms of MRONJ. Patients with a low risk of MRONJ can undergo all dental procedures as usual [14]. Patients with an increased MRONJ risk can still undergo non-invasive dental procedures when necessary but invasive procedures require expert advice [1, 2]. If carried out in accordance with established guidelines, invasive dental procedures, such as a tooth extraction, can be safely performed even in high-risk patients (those receiving BMA in an oncology setting). Low rates of MRONJ have been reported following tooth extraction in a patient population that included those receiving high-dose BMA for malignant disease [41]. Furthermore, it has been speculated that if a tooth extraction is performed to eliminate a local infection, and is performed with preventative measures (antibiotic prophylaxis and plastic wound closure), the procedure may actually decrease the long-term risk of MRONJ [30]. However, no randomized evidence currently supports such a hypothesis.

Barriers to successful MRONJ prevention can include poor patient knowledge or understanding of the recommended preventative strategies [42], which can be addressed by education. In a qualitative study of 23 patients with MRONJ, fear of visiting the dentist, lack of awareness of the importance of oral health, and cost of dental treatment were all identified as potential barriers to MRONJ prevention [42]. Oral health disparities, such as access to oral healthcare, the patient’s socioeconomic status, and health literacy, are also noted as barriers in the MASCC/ISOO/ASCO Clinical Practice Guideline [2]. At the healthcare-provider level, oncologists and dentists may not be aware of all BMA-related concerns when treating their patients. In an effort to increase awareness, Patel et al. developed a dental alert card, designed for patients on MRONJ-associated therapy, to show their dentist when undergoing assessment/treatment, notifying them of the patient’s underlying risk of MRONJ [43]. We propose an example dental alert card that could be adapted in Online resource 1; Supplementary data 6. Nurses should be mindful of potential barriers and ensure that patients are supported as far as possible in carrying out preventative measures and that appropriate information is exchanged between healthcare providers.

## Clinical management of MRONJ

If MRONJ is suspected, the patient should be referred to either an oral and maxillofacial surgeon, or a specialist oncology center with experience of MRONJ [21]. In the interim, a 0.12% chlorhexidine mouth rinse and/or amoxicillin with clavulanic acid 500/125 mg three times daily can be prescribed by a dentist or other qualified healthcare professional to treat any related infection (Fig. 2). Patients should be reassured that if MRONJ is diagnosed, the condition is treatable. They should be advised that the oral and maxillofacial surgeon will discuss the treatment options with them, and that this treatment may include surgery.

The AAOMS position paper has assigned treatment strategies according to the stage of MRONJ [14], the goals of which are to prioritize and support continued oncologic treatment and preserve quality of life through patient education and reassurance, and to control infection, progression of bone necrosis, and pain. For the initial treatment of MRONJ, the MASCC/ISOO/ASCO Clinical Practice Guidelines recommend non-surgical treatments, such as antimicrobial mouth rinses, effective oral hygiene, antibiotics if clinically indicated (i.e., when signs and symptoms of infection are present), and conservative surgical procedures [2], followed by aggressive surgical interventions for refractory cases. Notably, longer exposure time to bisphosphonates and more advanced stages of MRONJ significantly reduce MRONJ healing rates with non-surgical treatments [44]; therefore, it is important to identify and treat MRONJ early. While non-surgical interventions may be appropriate or necessary in certain situations [45–47] (for example, if the aim of therapy is a reduction of symptoms, or if the patient has no symptoms, or has severe symptoms but does not qualify for surgery), mucosal healing is rarely achieved with non-surgical treatments alone [45, 46], and with regard to this outcome, surgery may be more effective [13].

Surgical approaches to treatment include jaw resection, invasive sequestrectomy, and debridement [48]. Surgery may stop disease progression and lead to complete mucosal healing, with success rates of over 90% reported with certain procedures [45, 47], but the criteria for selecting a patient for surgery are not always agreed upon and require further study. The aim of surgery is to completely remove necrotic bone while avoiding unnecessary removal of healthy bone. Determining the precise margin of necrosis is challenging. Imaging techniques such as bone fluorescence may help to distinguish necrosis from healthy areas, improving the accuracy of surgical procedures [45, 47]. While surgery allows tissue samples to be extracted for histopathological evaluation and confirmation of MRONJ and/or metastasis, the results almost invariably show necrotic bone without impact on further patient management.

In contrast with the MASCC/ISOO/ASCO recommendations, a 2019 European task force working group on MRONJ concluded that surgical treatment is superior to non-surgical management and also suggested that early surgery for localized disease may be considered to prevent progression [17]. Surgical treatment may be superior to non-surgical interventions in terms of its predictability of duration.

Many other treatment strategies for MRONJ have been proposed. A systematic review of low-intensity laser, hyperbaric oxygen, and platelet-rich plasma concluded that these approaches are well tolerated [49], but there is a lack of evidence from randomized clinical trials [49–51]. Further well-designed randomized clinical trials are urgently needed to identify the best treatment approach.

## A multiprofessional approach to care and organization of the care pathway

**Fig. 3:**
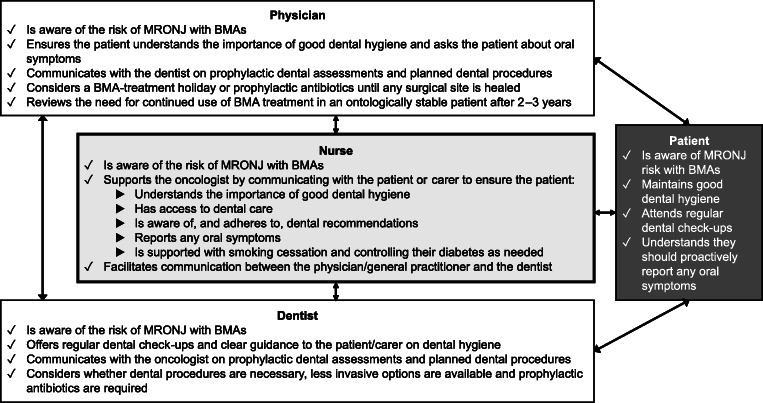
Multiprofessional approach to preventing and managing MRONJ. *BMA*, bone-modifying agent; *MRONJ*, medication-related osteonecrosis of the jaw

A multiprofessional team, involving nurse practitioners, dentists, physicians, oral oncologists, and oral and maxillofacial surgeons, is important to manage and treat MRONJ [2, 52]. Figure 3 illustrates a multiprofessional approach to preventing and managing MRONJ. The nurse has a role to play in managing the patient’s fears and concerns about MRONJ, promoting compliance with preventative measures and treatment, and facilitating communication between the patient, the physician, and the dentist. Lack of cooperation between dentists and physicians may adversely impact the incidence of MRONJ in osteoporosis treatment [53], highlighting an important area in which nurses can assist.

The nurse can also assist in decision-making on further BMA treatment. For example, shared decision-making plays a role when considering “drug holidays.” A systematic review of MRONJ treatment found that completely healed sites were significantly more common in patients wh6o had undertaken a BMA “drug-holiday,” but this has not been studied in a randomized trial [54]. In addition, there is only limited evidence of benefit for discontinuing BMAs before dentoalveolar procedures, and discontinuing BMAs may increase the risk of SREs and fracture [2]. Therefore, the MASCC/ISOO/ASCO Clinical Practice Guidelines leave this decision to the treating clinicians, recommending that dental specialists are consulted about the risk of MRONJ and oncologists are consulted about potential morbidity as a result of BMA discontinuation [2]. Nurses can play a role in encouraging communication between the physician and dentist, and ensuring that the patient understands the risks and benefits of the approach. Nurses may also be able to help improve the overall organization of the care pathway for patients with suspected MRONJ. For example, nurses might play a role in arranging quick access to an oral and maxillofacial surgeon for a patient who presents with suspicion of MRONJ. They might also promote communication with general practitioners and dentists and act to ensure that all healthcare professionals convey a unified message regarding benefit–risk profiles of BMAs.

## Conclusions

MRONJ occurs in approximately 1–9% of patients with advanced cancer exposed to high-dose BMAs [2]. Though infrequent, MRONJ is a serious adverse event that can cause significant pain and reduced quality of life, and can be difficult to treat [1–3]. MRONJ is a multiprofessional issue; nurses and other allied healthcare professionals have an important role to play in identifying MRONJ, assessing risk, counseling patients on the benefit–risk of BMAs, and preventing and managing MRONJ. Key practice points highlighted in the review are summarized in Table 3. Healthcare practitioners are urged to enhance their collaboration with one another on a local level to create dedicated care pathways that extend beyond counseling the individual patient.

**Table 3:** Key practice points identified by this review article for nurses and healthcare professionals in the identification, prevention, and management of MRONJ

1. To enable detection of MRONJ as early as possible, nurses can proactively ask the patient about the possible signs and symptoms of MRONJ (Fig. 1), and if there is any doubt, nurses should discuss their concerns with the treating physician, dentist, and general practitioner. 2. To assess risk factors for MRONJ, nurses can ask the patient questions based on the known risk factors for MRONJ and determine whether the patient is at low or increased risk of MRONJ (Online resource: Supplementary data 1/2). 3. To ensure patient compliance with MRONJ preventative measures and any subsequent treatment, nurses can provide the patient with clear explanations, information, and education on MRONJ risks and the preventative steps that can be taken to reduce this risk (Figs. 2 and 3). 4. To put the risk of MRONJ into context with the patient’s other health issues, nurses can educate the patient on how infrequent MRONJ is relative to the benefits of BMA treatment. 5. To ensure appropriate clinical management of MRONJ, nurses can encourage communication between the patient, the physician, and the dentist (Fig. 3).	

*BMA*, bone-modifying agent; *MRONJ*, medication-related osteonecrosis of the jaw

## Electronic supplementary material

ESM 1 (DOCX 377 kb)
