# Supplementary material for: Preventing, identifying, and managing medication-related osteonecrosis of the jaw: a practical guide for nurses and other allied healthcare professionals
Source: Support Care Cancer. 2020 Apr 19;28(9):4019–29. doi: 10.1007/s00520-020-05440-x (PMC7378104; doi:10.1007/s00520-020-05440-x)
Supplement: Supplementary file 1 — (DOCX 377 kb) [file 520_2020_5440_MOESM1_ESM.docx]

**Online resource 1 for: Preventing, identifying and managing medication-related osteonecrosis of the jaw: a practical guide for nurses and other allied healthcare professionals**

Lawrence Drudge-Coates,^1*^ Tim Van den Wyngaert,^2^ Morten Schiødt,^3^ H.A.M. van Muilekom,^4^ Gaston Demonty,^5^ Sven Otto^6^

*Author affiliations:* ^1^Department of Urology, King’s College Hospital NHS Foundation Trust, London, UK; ^2^Department of Nuclear Medicine, Antwerp University Hospital, Edegem, Belgium and Faculty of Medicine and Health Sciences, University of Antwerp, Antwerp, Belgium; ^3^Department of Oral and Maxillofacial Surgery, Rigshospitalet, Copenhagen, Denmark and Department of Clinical Epidemiology, Aarhus University Hospital, Aarhus, Denmark; ^4^Department of Urology, Antoni van Leeuwenhoek Hospital–Netherlands Cancer Institute, Amsterdam, Netherlands; ^5^Medical Development, Amgen (Europe) GmbH, Rotkreuz, Switzerland; ^6^Department of Oral and Maxillofacial Surgery, Ludwig-Maximilians-University of Munich, Munich, Germany

***Correspondence**: Lawrence Drudge-Coates, Department of Urology, King’s College Hospital NHS Foundation Trust, Denmark Hill, London SE5 9RS, UK; e-mail [ldrudge-coates@nhs.net](mailto:ldrudge-coates@nhs.net)

**Supplementary data 1** Assessing MRONJ risk in patients before starting a bone-modifying agent – questions to ask patients

| **Questions to ask your patients before starting bone-modifying agents** | **Yes** | **No** |
| --- | --- | --- |
| Have you previously been treated with bone-modifying agents, such as bisphosphonates or denosumab?  ***Additional considerations:***   - *What dosing regimen of bone-modifying agents was used?* - *For how long was the patient treated with bone-modifying agents?* |  |  |
| Have you previously been treated with any of the following?   - Corticosteroids - Chemotherapy - Treatments for cancer such as angiogenesis inhibitors |  |  |
| Have you previously received radiotherapy to the head or neck? |  |  |
| Do you have any pre-existing dental disease?  Have you had any serious gum infections such as periodontitis?  Have you had any dental surgery such as tooth extraction, insertion of dental implants or any other surgery in the region of the mouth?  If you wear dentures, have you had any problems with how they fit?  ***Additional considerations:***   - *Is the patient registered with a dentist?* - *When did the patient last see the dentist?* - *Has their dentist told them they may need future dental surgery?* - *Does the patient have any tooth pain or loose teeth at the moment?* |  |  |
| Do you smoke? |  |  |
| Do you have any of the following conditions/diseases?   - Cancer - Blood diseases (hematologic disease) - Immune system disorders - Diabetes mellitus - Anemia - Kidney problems   ***Additional consideration:***   - *Is the patient over 60 years old?* |  |  |

MRONJ, medication-related osteonecrosis of the jaw

**Supplementary data 2** A flow chart to determine MRONJ risk before a patient starts a bone-modifying agent [[1-11](#_ENREF_1)]


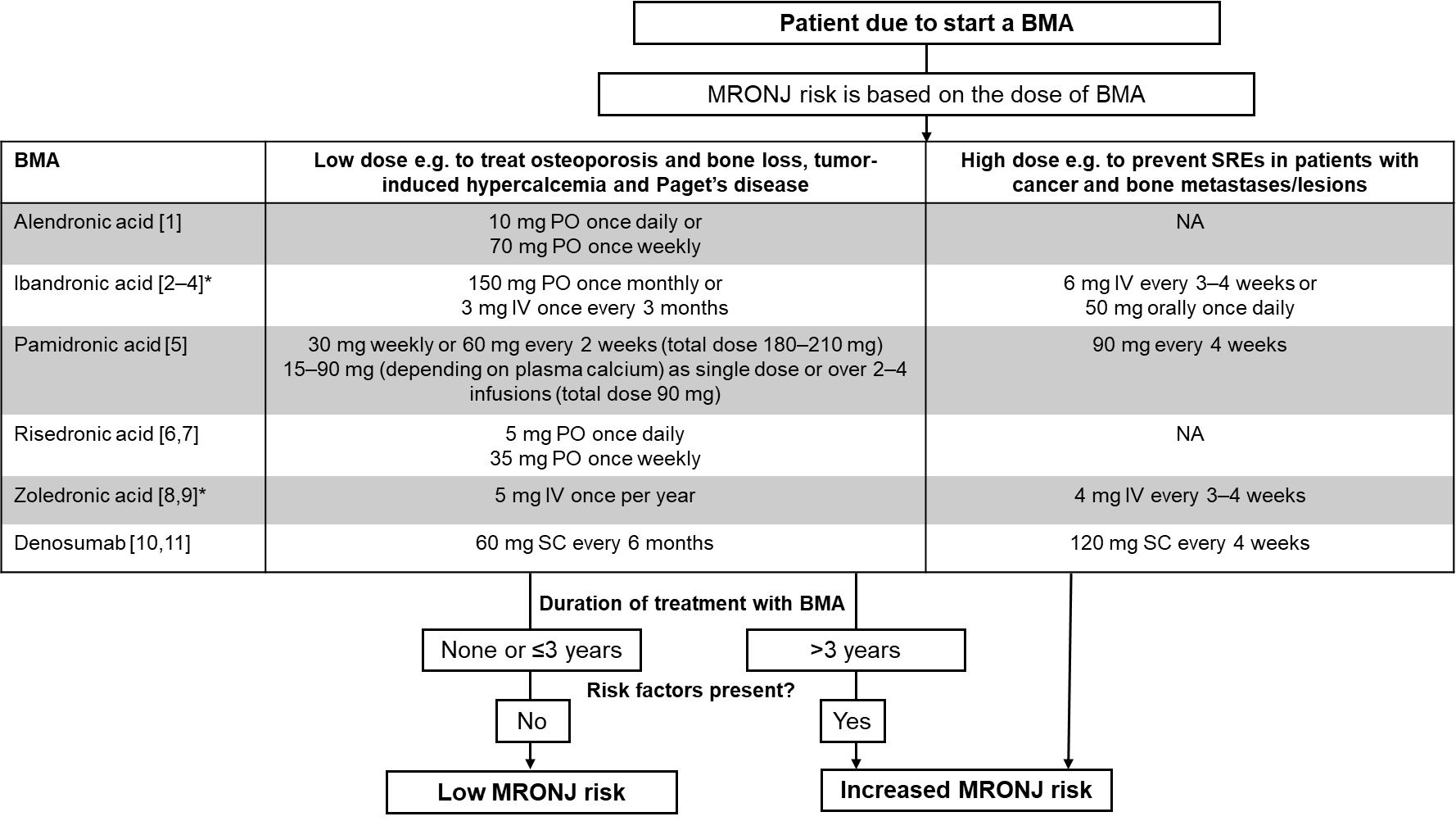


*Several versions of ibandronic acid and zoledronic acid are available

BMA, bone-modifying agent; IV, intravenous; MRONJ, medication-related osteonecrosis of the jaw; NA, not available; PO, per os; SC, subcutaneous; SRE, skeletal-related event

**Supplementary data 3** Likelihood of help versus harm during treatment with denosumab

The likelihood of help versus harm (LHH) during the first two years of starting denosumab therapy was calculated at approximately 17 in the average woman with breast cancer that has spread to the bones and who values the harm from a bone complication and medication-related osteonecrosis of the jaw (MRONJ) on the same level, using the following formula [[12](#_ENREF_12)]:

(1/NNT) * Ft * S

LHH = –––––––––––––––––––––––– = ~ 17

(1/NNH) * Fh

Based on the following assumptions:

- Number needed to treat (NNT) = ~3, based on a skeletal-related event rate of 64% with placebo [[13](#_ENREF_13)] and ~29% with denosumab [[14](#_ENREF_14)]
- Ft (patient’s risk of bone complication relative to trial population) = 1
- S (patient’s perception of severity of bone complication relative to MRONJ) = 1
- Number needed to harm (NNH) = 50 [[15](#_ENREF_15)]
- Fh (patient’s risk of MRONJ relative to trial population) = 1

**Supplementary data 4** Example of a tool that can be used to explain risk to patients [[16](#_ENREF_16)]

**
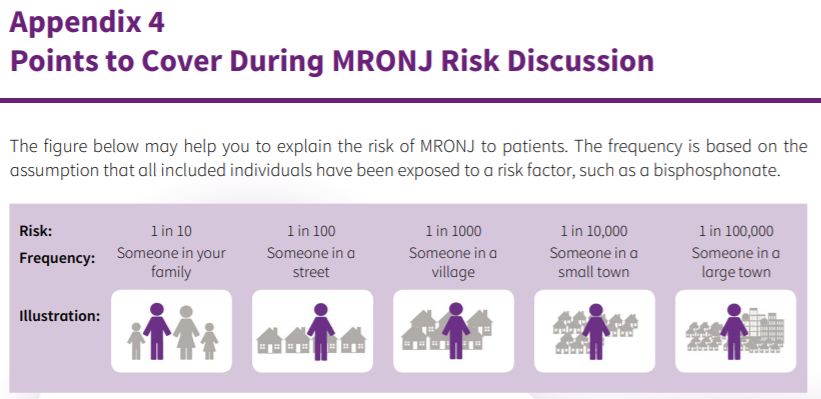
**

MRONJ, medication-related osteonecrosis of the jaw

Reproduced from Scottish Dental Clinical Effectiveness Programme (2017) Oral health management of patients at risk of medication-related osteonecrosis of the jaw: Dental clinical guidance <http://www.sdcep.org.uk/wp-content/uploads/2017/04/SDCEP-Oral-Health-Management-of-Patients-at-Risk-of-MRONJ-Guidance-full.pdf>. Accessed 27 February 2020

**Supplementary data 5** Example letter and information sheets to send to the patient’s dentist to explain that the patient will be starting a bone-modifying agent and that a dental evaluation is required

| Name  Hospital Address  Dear Dentist  Patient name:  Date of referral:  Diagnosis:  Name of bone-modifying agent:  Other relevant oncological treatment (please circle): chemotherapy / corticosteroids / antiangiogenic therapy  The above patient was seen recently in the [name of clinic] and a decision has been made to commence [insert name of bone-modifying agent] as part of their oncological treatment.  Medication-related osteonecrosis of the jaw (MRONJ) is a rare but serious complication that has been associated with the use of this drug, and so **we would be grateful if you could carry out a dental assessment on this patient prior to the commencement of [insert name of bone-modifying agent] therapy.**  Once [insert name of bone-modifying agent] therapy has started, we recommend that patients receive 6-monthly dental assessments.  It is believed that undergoing invasive dental procedures once on [insert name of bone-modifying agent] therapy significantly increases the risk of developing MRNOJ. For this reason, if the initial dental assessment indicates the need for dental extractions, these should be performed prior to starting the [insert name of bone-modifying agent] therapy and at least four weeks allowed for the socket to heal. If a dental extraction becomes necessary once on [insert name of bone-modifying agent] treatment, specialist management will be required. Hence, please refer your patients to your local Oral and Maxillofacial Unit.  Enclosed are guidelines for the dental health of oncology patients on bone-targeting agent therapy.  If you have any questions, please contact:  Name:  Title:  Phone number: |
| --- |

**Dental health guidelines for oncology patients**

| **SIGNS AND SYMPTOMS OF  MEDICATION-RELATED OSTEONECROSIS OF THE JAW (MRONJ)**   1. Absent or delayed hard and soft tissue healing after dental extractions 2. An area of exposed non-vital bone 3. Gingival and mucosal tissues surrounding necrotic bone usually inflamed and tender 4. Severe pain from secondary infection of necrotic bone 5. Paresthesia due to peripheral nerve compression secondary to acute infection of soft tissue 6. Microfractures resulting in sharp edges, which traumatize surrounding soft tissues and can cause constant pain 7. The necrotic process can spread if adjacent teeth are affected by periodontal disease 8. May be asymptomatic   **If MRONJ is ever suspected,  please refer the patient to your local Oral and Maxillofacial Unit** |
| --- |

| **THE DENTAL ASSESSMENT**  **Prior to a patient commencing bone-modifying agent therapy,  dental assessment should comprise:**   1. Comprehensive extra-oral and intra-oral examination 2. Radiographic assessment of teeth including orthopantomogram and long cone periapical radiographs, as clinically necessary 3. Identify and control any periodontal disease and dental caries 4. Evaluation of third molars 5. Perform any necessary extractions as soon as possible 6. Ensure dentures are atraumatic & comfortable 7. Eliminate sharp edges of teeth or restorations 8. Scaling of teeth and oral hygiene instruction 9. Arrangement of regular review of dental health |
| --- |

| **DENTAL CARE OF PATIENTS RECEIVING** **BONE-MODIFYING AGENT THERAPY**  **All patients should have oral hygiene instruction**  **Permitted treatments**  To be performed as atraumatically as possible:   - Routine restorations - Placement/replacement of crowns and bridges - Use of local anesthesia as necessary - Root canal treatment - Scaling and root planing (antibiotic prophylaxis may be necessary)   **Procedures to be avoided without prior expert advice**   - Dental extraction* - Oral/periodontal surgery that exposes or manipulates bone - Dental implants   ***Dental extraction in patients on bone-targeting agents should be performed with prophylactic measures and require specialist management. Hence, please refer the patient to your local Oral and Maxillofacial Unit** |
| --- |

**Supplementary data 6** Example dental alert card

**
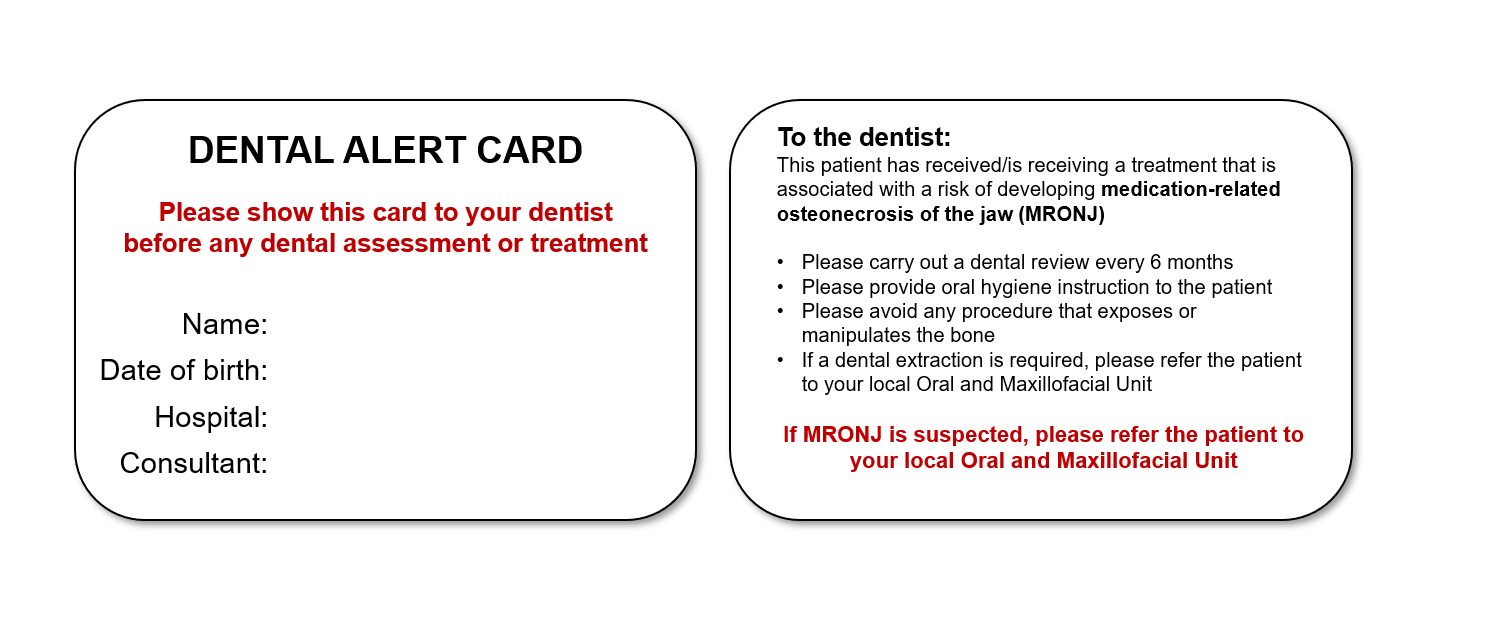
**

**Reference list**

1. Accord Healthcare Limited (2017) Alendronic acid summary of product characteristics. <https://www.medicines.org.uk/emc/product/6050/smpc>. Accessed 27 February 2020

2. Roche Registrations Limited (2019) Bonviva (ibandronic acid) summary of product characteristics. <https://www.ema.europa.eu/en/documents/product-information/bonviva-epar-product-information_en.pdf>. Accessed 27 February 2020

3. Roche Registrations Limited (2019) Bondronat (ibandronic acid) summary of product characteristics. <https://www.ema.europa.eu/en/documents/product-information/bondronat-epar-product-information_en.pdf>. Accessed 27 February 2020

4. Sandoz GmbH (2016) Ibandronic acid Sandoz: summary of product characteristics. <https://www.ema.europa.eu/en/documents/product-information/ibandronic-acid-sandoz-epar-product-information_en.pdf>. Accessed 27 February 2020

5. Wockhardt UK Ltd (2017) Disodium pamidronate; summary of product characteristics. <https://www.medicines.org.uk/emc/medicine/21443>. Accessed 27 February 2020

6. Aurobindo Pharma - Milpharm Limited (2018) Risedronate sodium summary of product characteristics. <https://www.medicines.org.uk/emc/medicine/27563>. Accessed 27 February 2020

7. Warner Chilcott (2018) Actonel (risedronic acid) summary of product characteristics. <https://www.medicines.org.uk/emc/medicine/3341>. Accessed 27 February 2020

8. Novartis Pharma GmbH (2018) Zometa (zoledronic acid) summary of product characteristics. <https://www.ema.europa.eu/en/documents/product-information/zometa-epar-product-information_en.pdf>. Accessed 27 February 2020

9. Novartis Europharm Limited (2019) Aclasta (zoledronic acid) summary of product characteristics. <https://www.ema.europa.eu/en/documents/product-information/aclasta-epar-product-information_en.pdf>. Accessed 27 February 2020

10. Amgen Europe B. V. (2019) Prolia (denosumab) summary of product characteristics. <https://www.ema.europa.eu/en/documents/product-information/prolia-epar-product-information_en.pdf>. Accessed 27 February 2020

11. Amgen Europe B. V. (2019) Xgeva (denosumab) summary of product characteristics. <https://www.ema.europa.eu/en/documents/product-information/xgeva-epar-product-information_en.pdf>. Accessed 27 February 2020

12. Guyatt G (2008) Users’ Guides to the Medical Literature: A Manual for Evidence-Based Clinical Practice. McGraw-Hill, New York

13. Lipton A, Theriault RL, Hortobagyi GN, Simeone J, Knight RD, Mellars K, Reitsma DJ, Heffernan M, Seaman JJ (2000) Pamidronate prevents skeletal complications and is effective palliative treatment in women with breast carcinoma and osteolytic bone metastases: long term follow-up of two randomized, placebo-controlled trials. Cancer 88:1082-1090

14. Stopeck A, de Boer R, Fujiwara Y, Lichinitser M, Tonkin K, Yardley D, Fan M, Jiang Q, Jun S, Dansey R, Braun A (2009) A Comparison of Denosumab Versus Zoledronic Acid for the Prevention of Skeletal-Related Events in Breast Cancer Patients with Bone Metastases. Cancer Research 69:22-22

15. Kyrgidis A, Toulis KA (2011) Denosumab-related osteonecrosis of the jaws. Osteoporos Int 22:369-370

16. Scottish Dental Clinical Effectiveness Programme (2017) Oral health management of patients at risk of medication-related osteonecrosis of the jaw: Dental clinical guidance <http://www.sdcep.org.uk/wp-content/uploads/2017/04/SDCEP-Oral-Health-Management-of-Patients-at-Risk-of-MRONJ-Guidance-full.pdf>. Accessed 27 February 2020
